# Physiological responses and balance ability are affected by physical workload and heat exposure in workers

**DOI:** 10.1186/2046-7648-4-S1-A25

**Published:** 2015-09-14

**Authors:** Su-Young Son, Ken Tokizawa, Akinori Yasuda, Chin-ichi Sawada

**Affiliations:** 1 National Institute of Occupational Safety and Health, Kawasaki, Japan

## Introduction

Japan is experiencing high temperatures and relative humidity (rh) during the summer months, which lead to increases in the incidence of heat stroke among outdoor workers. According to the Ministry of Health, Labour and Welfare in Japan, between 2010 and 2012, approximately 86 deaths were caused by heat stroke, of which 40% occurred at construction sites [1]. This study aimed to determine whether physical performance, especially balance ability, deteriorates with increased physical workload and heat exposure. In addition, we aimed to determine the relationship of physiological responses and balance function with heat exposure.

## Methods

Ten healthy males were included in the study (mean (SD): age 31.9(7.9) yrs; weight, 64.6(6.7) kg; height, 171(4.5) cm). The participants were required to wear a work suit, helmet, gloves, and boots. The test procedures included a 1-hr workload test (2 × 25 min exercise bouts walking at 5.5 km.h^-1^, separated by 10 min) with 1-hr recovery period, body sway test, and functional reach test. The test chamber was maintained at 27 °C and 40% rh for the Low temperature (LT) condition, 37 °C and 70 % rh for high temperature (HT) condition. Two types of balance ability tests were performed before and after the workload test and during the recovery period. The first was the postural balance test and the second was the functional reach test. All of the experimental procedures were approved by the Human Research Ethics Committee of our Institute.

## Results

Rectal temperature (T_re_) increased during the workload test, with significant differences between the LT and HT conditions in the recovery period. The increase in T_re _(ΔT_re_) under the LT and HT conditions were 0.5(0.1) °C and 1.0(0.2) °C, respectively (p < 0.001). The mean body weight loss was significantly greater under the HT condition than under the LT conditions (1.0(0.3) kg vs. 0.5(0.2) kg, p < 0.001). In the functional reach test, the mean values decreased with workload and were lower at the middle of the recovery period. However, no significant differences were observed between the LT and HT conditions. Body sway length and sway area were greater under the HT condition than the LT condition. In addition, these values increased with workload and decreased during the recovery period. For the eyes closed trials, the body sway parameter values increased at the middle of the recovery period. However, no significant differences were observed.

## Discussion

The main finding of this study shows that physiological responses and balance ability were affected by physical workload and heat exposure. The physiological responses, ΔT_re_, and body weight loss were significantly higher under the HT condition than under the LT condition. Even though body sway parameters did not shown significant differences between two conditions, it tended to increase with workload or heat exposure. Moreover, body balance ability deteriorated more until the middle of the recovery period with heat exposure and could not be recovered enough during the recovery period.

## Conclusion

In conclusion, this study demonstrated that physiological responses and balance ability are affected by physical workload and heat exposure. Based on our results, less than 30 min of the recovery period is seems to contribute to the occurrence of adverse events after physical workload in a hot environment.
